# Effects of Physical Exercise, Cognitive Stimulation, and Their Combination on Mobility, Cognitive Function, and Psychosocial Well-Being in Older Adults Residing in Long-Term Care: Protocol for a Randomised Controlled Trial

**DOI:** 10.3390/jcm15166327

**Published:** 2026-08-16

**Authors:** Nuno Carvalho, Rocío Llamas-Ramos, Inés Llamas-Ramos

**Affiliations:** 1 Doctoral Programme in Health, Disability, Dependency and Well-Being (Salud, Discapacidad, Dependencia y Bienestar), Universidad de Salamanca, 37007 Salamanca, Spain; nuno.carvalho@usal.es; 2 Insight: Piaget Research Center for Ecological Human Development, Instituto Piaget, 1950-157 Lisbon, Portugal; 3 Department of Nursing and Physiotherapy, Universidad de Salamanca, 37007 Salamanca, Spain; inesllamas@usal.es; 4 Instituto de Investigación Biomédica de Salamanca (IBSAL), 37007 Salamanca, Spain; 5 University Hospital of Salamanca, 37007 Salamanca, Spain

**Keywords:** aged, long-term care, exercise, cognitive training, executive function, mobility limitation, quality of life, clinical trial protocol

## Abstract

**Background/Objectives**: Older adults residing in long-term care frequently experience multimorbidity, low habitual activity, mobility decline, executive dysfunction, and reduced quality of life. Evidence separating the effects of exercise, cognitive stimulation, and their combination remains limited. The primary objective of this trial is to determine whether a 12-week combined physical exercise plus cognitive stimulation programme improves executive function, functional mobility, and health-related quality of life compared with usual care. Secondary objectives are to estimate the effects of each single-component programme, compare the combined programme with each component, and examine maintenance at six months. **Methods**: This is a four-arm, parallel-group, assessor-blinded randomised controlled trial in Portuguese long-term care facilities. A total of 140 residents aged 65 years or older, residing in long-term care for at least three months and able to provide informed consent, will be allocated 1:1:1:1 to supervised multicomponent exercise, group cognitive stimulation, combined exercise plus cognitive stimulation, or usual care. Active programmes will be delivered three times weekly for 12 weeks. Assessments are planned at baseline, post-intervention, and six-month follow-up according to the prespecified schedule. Primary outcomes are the Stroop interference score, Timed Up and Go time, and EQ-5D-5L index value at 12 weeks. Analyses will follow the intention-to-treat principle using mixed-effects models; multiplicity across the three primary endpoints will be controlled using Holm’s procedure, and interaction analyses will be exploratory and hypothesis-generating. **Results**: Recruitment and data collection had not started at manuscript submission; therefore, no participant data are reported. **Conclusions**: The trial will estimate the effects of the combined programme versus usual care and provide exploratory comparative evidence on the single-component programmes in real-world long-term care.

## 1. Introduction

Residents of long-term care facilities commonly experience accelerated functional and cognitive decline. Multimorbidity, polypharmacy, sedentary routines, and restricted opportunities for autonomous activity can reduce strength, balance, mobility, participation, and health-related quality of life. Executive abilities such as planning, inhibition, and divided attention are important for safe transfers, adapting gait, following daily routines, and engaging in meaningful activities. Decline in these domains is associated with greater dependence, falls risk, and poorer quality of life [1,2,3]. Participation in health-promoting activities also depends on the interaction between individual capability, motivation, social opportunity, and the physical environment, as emphasised by the COM-B framework and multidimensional active-ageing models [4,5]. Interventions must therefore be safe, accessible, motivating, compatible with facility routines, and sufficiently standardised for replication.

Supervised multicomponent physical exercise is a core non-pharmacological strategy for older adults. Programmes that combine balance, strength, flexibility, and functional mobility are consistent with international guidance and current geriatric exercise recommendations [6,7,8,9,10]. In long-term care, exercise can improve physical function and reduce factors associated with falls [2,3]. It may also support cognitive health through better vascular and metabolic function, sleep, mood, and brain plasticity [11,12,13]. These potential benefits are relevant where inactivity and limited environmental stimulation can accelerate both motor and cognitive decline.

Cognitive stimulation offers a complementary pathway through structured group activities involving attention, memory, executive control, language, orientation, calculation, and problem-solving. Such activities can support cognitive engagement and social interaction [14,15,16]. However, the strongest evidence for formal cognitive stimulation therapy concerns people with dementia [14,15]; evidence for group cognitive stimulation in older adults without a major neurocognitive disorder is smaller and partly extrapolated from cognitive-training research [16]. Testing this extrapolation is therefore an important purpose of the present trial. A combined physical-cognitive programme may target both motor capacity and executive control within the same care pathway [17,18].

Evidence on combined programmes remains heterogeneous in intervention dose, content, professional resources, outcomes, follow-up, and comparator definition. Trials of multicomponent exercise with simultaneous cognitive training suggest possible benefits in selected executive domains [19], while small pilot work integrating physical activity with mindful awareness supports feasibility but cannot establish comparative effectiveness [20]. Most studies compare one programme with usual care, so they cannot distinguish exercise effects from cognitive-stimulation effects or determine whether a combined programme adds benefit beyond either component. Complex behavioural interventions also make participant and provider blinding impracticable and create challenges for intervention fidelity, contact-time matching, and implementation [21]. The present four-arm design addresses these challenges through common eligibility and assessment procedures, blinded outcome assessment, standardised intervention manuals, component-specific fidelity records, and direct comparisons of exercise alone, cognitive stimulation alone, their combination, and usual care under the same facility conditions.

The primary objective is to determine whether a 12-week combined physical exercise plus cognitive stimulation programme improves executive function (Stroop interference score), functional mobility (Timed Up and Go time), and health-related quality of life (EQ-5D-5L index value) at 12 weeks compared with usual care. Secondary objectives are to estimate the effects of exercise and cognitive stimulation separately, compare the combined programme with each single-component programme, examine maintenance of selected outcomes at six months, and describe safety, adherence, and feasibility. The principal hypothesis is that the combined intervention will produce greater improvements than usual care across the three primary domains. Comparisons involving the single-component arms and formal interaction analyses are prespecified as exploratory and hypothesis-generating.

## 2. Materials and Methods

### 2.1. Trial Design, Setting, and Reporting Framework

This is a pragmatic, randomised, parallel-group, assessor-blinded, four-arm controlled trial with a 1:1:1:1 allocation ratio. Participants will be allocated to multicomponent physical exercise, cognitive stimulation, combined physical exercise plus cognitive stimulation, or usual care. Assessments are planned at baseline (T0), immediately after the 12-week intervention period (T1), and six months after programme completion (T2). The trial is designed to preserve ecological validity by delivering interventions on site within the participating long-term care facilities rather than in a laboratory or hospital environment.

The study will be conducted in four long-term care facilities (Estruturas Residenciais para Pessoas Idosas, ERPI units) operated by three non-profit private social solidarity providers in the district of Viseu, Portugal, in collaboration with Escola Superior de Saúde Jean Piaget de Viseu. One provider operates two of the participating ERPI units. Written institutional authorisation has been obtained from all four ERPI units. At the providers’ request, their identities are not reported in the public manuscript. The manuscript follows SPIRIT 2025 as the primary protocol-reporting framework, with CONSORT 2025 and TIDieR elements used where relevant [22,23,24]. The SPIRIT checklist, TIDieR intervention description, bilingual Portuguese-English participant information sheet and informed consent form, and a separate high-resolution planned CONSORT-style flow diagram are provided as supporting files.

### 2.2. Trial Status, Ethics, Registration, and Participant Consent

**Figure 1:**
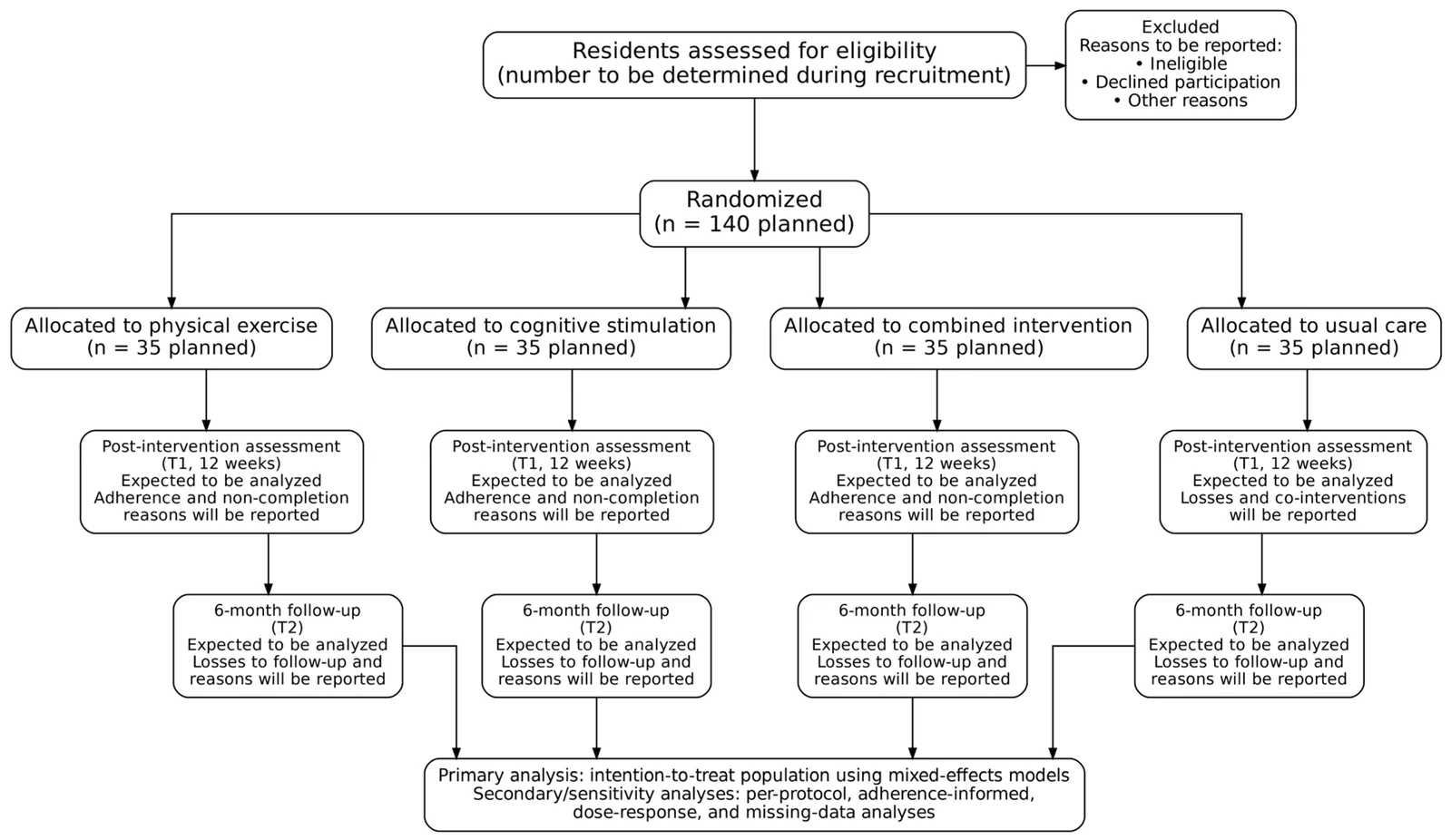
Planned CONSORT-style flow diagram for participant screening, allocation, post-intervention assessment, and 6-month follow-up.

Participant screening, recruitment, and data collection had not started at the time of manuscript submission. Written institutional authorisation has been obtained from all four participating ERPI units. Accordingly, no participant flow, baseline data, or trial outcomes are reported. This protocol describes the planned trial design, intervention procedures, assessment framework, and analysis strategy. The planned participant pathway is summarised in Figure 1.

This study is being conducted as part of the Doctoral Programme in Health, Disability, Dependency and Well-being at the University of Salamanca, in collaboration with Escola Superior de Saúde Jean Piaget de Viseu and the InSIGHT Research Centre, Instituto Piaget, Portugal. Within this collaborative framework, the participant information sheet and informed consent form underwent official translation into Portuguese and back-translation to support linguistic and conceptual equivalence for their use in Portugal. The trial received prospective ethical approval from the Research Ethics Committee of the University of Salamanca (registry no. 1379; 20 May 2025). At the protocol-planning stage, the Ethics Committee of the Piaget Institute (CEIP), Portugal, was consulted regarding the need for an additional ethical review in Portugal and indicated that no further ethical review was required in view of the existing prospective ethical approval from the Research Ethics Committee of the University of Salamanca. Written institutional authorisation to conduct the study was obtained from each of the four participating ERPI units before recruitment. No resident was approached before institutional authorisation was completed, and recruitment and study procedures will be initiated in accordance with the approved protocol and site procedures. The study is registered at ClinicalTrials.gov (NCT07082504). All procedures will comply with the Declaration of Helsinki, applicable Portuguese requirements, and the General Data Protection Regulation [25,26].

Written informed consent will be obtained before baseline testing from participants with full decision-making capacity. Participants will be informed that participation is voluntary, that they may withdraw without penalty, and that withdrawal will not affect facility care. Eligibility and consent procedures will be implemented in collaboration with facility technical directors and nursing teams. Screening will use resident lists, available clinical records, staff referral, and routine institutional assessment of the capacity to understand the study information and follow instructions. No biological specimens will be collected. General study results will be communicated to participants and participating facilities according to participants’ preferences and institutional procedures.

### 2.3. Patient and Public Involvement

No patients or members of the public were formally involved in drafting the original protocol. Nevertheless, the intervention and assessment procedures were selected to be feasible within routine long-term care, and operational input from participating facilities informed scheduling, safety procedures, and the choice of group-based delivery. During trial implementation, practical feedback from residents and staff regarding acceptability, session burden, and logistical feasibility will be documented to inform interpretation and future scaling.

### 2.4. Participants and Recruitment

Potential participants will be residents of the participating long-term care facilities. Inclusion criteria are age 65 years or older; residence in the facility for more than three months; capacity to provide informed consent and understand the study instructions; sufficient Portuguese literacy to complete the Torga Stroop test; and willingness to complete the planned assessments and intervention procedures. Cognitive exclusions are a documented major neurocognitive disorder or other impairment that precludes valid consent or comprehension of instructions. Physical and safety exclusions are an acute medical condition, an unstable cardiovascular or respiratory condition, an unresolved injury following a recent fall, a recent fracture or surgery that makes exercise unsafe, or a physical or sensory impairment that prevents safe completion of study procedures despite reasonable adaptation. A recent fall alone will not exclude a resident because falls risk is clinically relevant to the target population; unresolved injury or medical instability will prompt temporary ineligibility and medical review. Nursing staff and a physiotherapist will complete a pre-exercise safety screen, and medical clearance will be requested whenever contraindications are uncertain.

Recruitment will be conducted within the facilities through staff referral and screening of resident lists. Eligible residents will receive oral and written study information. Baseline assessment will include sociodemographic and clinical variables, medication use, length of residence, recent falls and fractures, exercise contraindications, and standardised physical, cognitive, and psychosocial measures. The numbers screened, excluded, randomised, assessed after intervention, and assessed at follow-up will be documented to support transparent reporting of feasibility and retention.

Recruitment in long-term care is influenced by clinical instability, fluctuating motivation, family involvement, and the operational routines of each institution. For this reason, the trial emphasises local coordination and resident-centred communication rather than external recruitment campaigns. Facility staff may support the identification of residents who can be approached, but participation will remain a research decision made by each eligible resident after receiving study information. This process is intended to balance feasibility with respect for autonomy and to reduce the risk that the resident-care relationship could be perceived as pressure to participate.

### 2.5. Randomisation, Allocation Concealment, and Blinding

Randomisation will be performed only after eligibility confirmation, written consent, and completion of baseline assessment. Participants will be allocated 1:1:1:1 within each ERPI using computer-generated lists with randomly varying block sizes. An adjunct professor at Escola Superior de Saúde Jean Piaget de Viseu who has no role in recruitment, baseline or outcome assessment, intervention delivery, or data analysis will generate and hold the allocation sequence. The sequence will be stored in a password-protected file on an institutional computer accessible only to this custodian. After baseline data are confirmed as complete, the facility technical director will send the coded participant identifier to the custodian, who will reveal only that participant’s assignment through individual secure communication. Local staff will retain the revealed assignment and intervention schedule but will not have access to future allocations. Outcome assessors will remain blinded. These safeguards distinguish allocation concealment before assignment from assessor blinding after assignment.

Participants and intervention providers cannot be blinded because of the behavioural nature of the programmes. Outcome assessors will be blinded to group allocation and will conduct assessments using participant lists that include identifiers but not treatment assignment. Participants and staff will be reminded not to disclose intervention participation during assessments. Statistical analysis will use coded group labels until the primary analyses are complete.

Contamination is a realistic risk in residential facilities because residents share social spaces and may discuss activities. To mitigate this, interventions will be scheduled separately where feasible, provider teams will follow predefined procedures, and usual-care activities will be documented monthly. Baseline comparability and any material changes in routine activities will be reported transparently, and sensitivity analyses will consider potential clustering or contamination effects.

### 2.6. Interventions

All active interventions will be group-based, delivered face-to-face on site, and scheduled three times per week for 12 weeks. Attendance, protocol deviations, and relevant occurrences will be recorded. The combined arm will receive both intervention components during the same visit, which reflects a realistic multimodal delivery model but introduces greater contact time than the single-component arms; this issue will be considered when interpreting comparative effects.

Table 1 summarises the main intervention components. More detailed intervention reporting is provided in Supplementary Material S2.

#### 2.6.1. Physical Exercise Programme

The physical exercise programme is designed to improve balance, strength, aerobic endurance, flexibility, and functional mobility while minimising risk and preserving feasibility in long-term care. Each approximately 60-min session will include about 50 min of active exercise and 10 min for transitions and individualised rest: 5 min of joint mobility and seated or standing marching; 10 min of balance training; 20 min of resistance and functional-mobility exercises; 10 min of continuous or interval walking or seated stepping; and 5 min of cool-down, breathing, and flexibility. Balance tasks will include supported static and dynamic postures, progressive reductions in base of support, controlled weight shifts, stepping, turning, figure-of-eight walking, and obstacle negotiation when clinically appropriate [6,7,8,9,10].

Resistance and functional exercises will include sit-to-stand, knee extension, plantar flexion, hip abduction, upper-limb pulling or elbow flexion, and step practice using body weight, chairs, elastic bands, bottles, or ankle weights. Weeks 1–2 will begin with one set of 8–10 repetitions; weeks 3–6 will progress toward two sets of 8–12 repetitions; and weeks 7–12 may progress to two or three sets of 8–12 repetitions according to tolerance. Exercise will be tailored to baseline function while preserving the standardised session structure. Aerobic intensity will be maintained at a positive talk test, allowing complete sentences with some effort, and overall exertion will start at 2–3 and progress to 3–4 on the Borg category-ratio 10 scale (CR10) [27,28,29]. Progression will be reviewed every two weeks and implemented only after two consecutive sessions are completed with safe technique, Borg CR10 no higher than 3 at the current dose, an appropriate talk test, and no clinically relevant symptoms. Only one variable will be increased at a time: repetitions up to 12, then the smallest available resistance increment, task complexity, or aerobic duration. Sessions will be paused or discontinued for chest pain, marked breathlessness, dizziness, symptomatic hypotension, new or worsening pain, gait instability, or any clinically relevant change in well-being, followed by nursing or medical review according to facility procedures.

#### 2.6.2. Cognitive Stimulation Programme

The cognitive stimulation programme will consist of small-group sessions designed to stimulate executive and broader cognitive domains relevant to daily functioning. Activities will include structured games, riddles, puzzles, orientation tasks, reasoning exercises, inhibition tasks, memory activities, and paper-and-pencil exercises targeting attention, working memory, cognitive flexibility, language, and visuospatial skills [14,15,16].

Tasks will be adapted according to participants’ comprehension and performance, progressing from simpler to more complex activities over the intervention period. Group delivery is intended not only to stimulate cognition but also to reinforce social interaction, motivation, and adherence, which are central to sustainable long-term care programming.

#### 2.6.3. Combined Intervention and Concomitant Care

Participants in the combined arm will receive both programmes during the same visit, with exercise followed immediately by cognitive stimulation. Attendance will be recorded separately for each component so that delivered dose can be characterised accurately. The design reflects a clinically plausible multimodal intervention but also introduces higher weekly structured contact time than the single-component arms; this will be acknowledged explicitly when interpreting whether the combined arm performs better than either single-component arm.

Routine facility care, regular medication, and usual supportive services will be permitted in all groups. New structured physiotherapy or organised exercise/sport programmes outside the study interventions will be discouraged when feasible and, if they occur, documented for sensitivity analyses. Monthly logs completed by facility technical directors will record relevant co-interventions, new rehabilitation programmes, and material changes in routine activities.

### 2.7. Outcomes and Assessment Schedule

The three primary outcomes are assessed at 12 weeks (T1): functional mobility measured by Timed Up and Go (TUG) time, with dual-task TUG as a supportive measure [30,31]; executive function measured by the Torga Stroop inhibitory-control index (correctly read words per second minus correctly named incongruent colours per second; lower values indicate better inhibitory control) [32,33]; and health-related quality of life measured by the EQ-5D-5L index value derived from the Portuguese value set [34,35]. The EQ visual analogue scale (EQ-VAS) will be collected at the same visits as a supportive quality-of-life measure.

Secondary outcomes include balance assessed with the Performance-Oriented Mobility Assessment (POMA/Tinetti) [36]; lower- and upper-limb strength, flexibility, and aerobic endurance assessed using Senior Fitness Test components, with handgrip strength assessed by dynamometry (Baseline® Hydraulic Hand Dynamometer (Fabrication Enterprise Inc., Elmsford, NY, USA) [37]; global cognition measured by the Mini-Mental State Examination (MMSE) and complementary executive measures using the Trail Making Test (TMT) and Digit Span [38,39,40]; sleep quality measured by the Pittsburgh Sleep Quality Index (PSQI) [41,42]; depressive symptoms, life satisfaction, and psychological well-being measured by the Geriatric Depression Scale (GDS), Satisfaction With Life Scale (SWLS), and Psychological Well-Being Scales (PWBS) [43,44,45]; functional independence measured by the Barthel Index [46]; physical activity measured by the Global Physical Activity Questionnaire (GPAQ) and pedometer monitoring (three-axis digital pedometer (Walking Style One 2.1, HJ-321-E; OMRON HEALTHCARE Co., Ltd., Muko, Kyoto, Japan) [47,48]; and anthropometrics, vital signs, and selected sociodemographic and clinical variables. Blood pressure and resting heart rate will be measured using an automated upper-arm blood pressure monitor (OMRON M6 Comfort, HEM-7221-E; OMRON Healthcare Co., Ltd., Kyoto, Japan) and body mass will be measured using a digital scale (Seca 813; seca GmbH & Co. KG, Hamburg, Germany), and height using a portable stadiometer (Seca 213; seca GmbH & Co. KG, Hamburg, Germany.

Trained assessors blinded to allocation will perform assessments according to the prespecified schedule. The three primary outcomes, the EQ-VAS, TMT, and Digit Span will be measured at baseline (T0), 12 weeks (T1), and six months after programme completion (T2). The MMSE and most other secondary outcomes will be measured at T0 and T1 only, as shown in Table 2 and Table 3. Written standard operating procedures will standardise administration, scoring, and data recording.

The EQ-5D-5L and EQ-VAS will be resident-reported and administered by a trained blinded assessor using the standardised Portuguese interviewer-administered format. The assessor will read the standard instructions and items and record the resident’s own responses without interpretation or prompting. For frail residents with physical, visual, hearing, or fatigue-related difficulties, usual sensory aids, additional time, and rest breaks will be permitted. Proxy responses will not be used; if a resident is unable to provide a valid response despite these accommodations, the corresponding measure will be recorded as missing.

### 2.8. Sample Size

The target sample size is 140 randomised participants (approximately 35 per arm). This is the pragmatic recruitment target rather than a zero-dropout analysable sample; loss of up to 20% by T1 is anticipated, leaving approximately 112 participants (28 per arm). The sample is feasibility-driven, reflecting the recruitment capacity of the participating facilities and intervention workload. The Torga validation study reported a standard deviation of 0.43 for its inhibitory-control index [33]. With 35 participants in the combined and usual-care arms, a two-sided independent comparison at alpha = 0.05 and 80% power has a minimum detectable difference of approximately 0.67 standard deviations, equivalent to 0.29 index points. If 20% are unavailable at T1 (28 per arm), the corresponding value is approximately 0.75 standard deviations or 0.32 index points. Baseline-adjusted mixed models may improve precision, but these conservative values make clear that the trial is designed to detect moderate-to-large rather than small effects.

The primary estimand is the adjusted between-group difference in change from baseline to T1 for the combined intervention versus usual care. This estimand will be evaluated for the Stroop interference score, TUG time, and EQ-5D-5L index. Effect estimates and 95% confidence intervals will be emphasised. Comparisons involving the exercise-only and cognitive-stimulation-only arms, maintenance at T2, and exercise-by-cognitive-stimulation interaction effects are exploratory because the pragmatic sample was not selected to power those tests.

### 2.9. Data Management and Quality Assurance

Data will be recorded on standardised collection forms and then entered into a protected electronic database accessible only to authorised members of the research team. Participant identifiers will be stored separately from outcome data, and each participant will be assigned a study code. Data checks will include verification of missing fields, range plausibility, and consistency across time points. Only de-identified data will be used for analysis and reporting.

Fidelity procedures will include provider training before intervention delivery, use of session templates and attendance logs, and documentation of relevant deviations or unusual events. Written standard operating procedures will be used to support consistency in assessment, intervention progression, and adverse-event recording. Any protocol amendments affecting eligibility, outcomes, or procedures will be documented and communicated to the participating institutions and research personnel before implementation.

### 2.10. Statistical Analysis

Statistical analyses will be performed using IBM SPSS Statistics, version 32.0 (IBM Corp., Armonk, NY, USA). Analyses will follow the intention-to-treat principle, with all randomised participants analysed in their allocated groups regardless of adherence [49]. Baseline characteristics will be summarised using appropriate descriptive statistics. Mixed-effects models will estimate changes over time and group-by-time differences for each outcome distribution [50]. Models will include group, time, group-by-time interaction, baseline outcome value, and facility as a fixed effect or clustering factor, as supported by the number of participating facilities and model stability.

The primary family comprises the combined-intervention versus usual-care contrasts at T1 for the Stroop interference score, TUG time, and EQ-5D-5L index. The family-wise two-sided Type I error rate will be controlled at 0.05 using Holm’s step-down procedure. Secondary between-arm contrasts and secondary outcomes will be treated as exploratory; within prespecified outcome families and time points, false discovery rate will be controlled using the Benjamini–Hochberg procedure, with both unadjusted and adjusted p-values reported. Follow-up models will examine maintenance only for outcomes collected at T2. Supportive analyses may include per-protocol, adherence-informed, dose-response, and co-intervention-adjusted models. Multiple imputation may be used if missingness is non-trivial and the assumptions are defensible [51] (Table 4).

Results will be reported as adjusted mean differences or appropriate model-based contrasts with 95% confidence intervals. The direction of benefit will be stated explicitly. Adherence will be summarised by arm and component using sessions attended, percentage of planned sessions completed, and total minutes delivered. A factorial coding of exercise (present/absent) and cognitive stimulation (present/absent) will be used in an exploratory exercise-by-cognitive-stimulation-by-time model. Because the trial is not powered for interaction testing and the combined arm has greater contact time, these analyses will be described as hypothesis-generating and will not support confirmatory claims of synergy.

### 2.11. Safety Monitoring and Adverse Events

The interventions are considered low risk, but adverse events may include fatigue, transient pain, dizziness, hypotension, fear of falling, emotional discomfort during cognitive tasks, or exacerbation of pre-existing symptoms. During assessments and intervention sessions, staff will monitor participants for signs of discomfort or instability. If clinically indicated, sessions will be interrupted and institutional or medical follow-up will be initiated according to local procedures.

All adverse events and unusual occurrences will be recorded, together with their timing, severity, management, and whether they appear related to study procedures. Because this is a behavioural intervention protocol conducted in residential care rather than a drug trial, no formal data monitoring committee has been convened. Oversight will be exercised by the principal investigators and collaborating institutions, with transparent reporting of harms in the final trial report.

### 2.12. Trial Status

At the time of manuscript submission, participant recruitment, baseline assessments, intervention delivery, and follow-up had not yet started. Consequently, the flow diagram provided in Figure 1 is presented as a planned CONSORT-style diagram, and all references to screening numbers, allocation totals, retention, and outcome data are intentionally omitted from the present manuscript.

## 3. Discussion

This protocol describes a pragmatic four-arm randomised trial addressing a practical long-term care question: whether a combined physical exercise plus cognitive stimulation programme improves executive function, mobility, and health-related quality of life compared with usual care, and how the estimates from exercise-only and cognitive-stimulation-only programmes compare. The design extends a simple intervention-versus-control trial by placing all four strategies under common eligibility, assessment, and facility conditions. This component-specific information is relevant because the programmes differ in professional requirements, materials, scheduling demands, participant preferences, and implementation costs.

The intervention model is clinically plausible: multicomponent exercise targets capacities required for transfers, walking, and fall prevention, while cognitive stimulation targets attention, inhibition, working memory, and problem-solving relevant to daily function. The combined programme may produce broader effects, but greater weekly contact time prevents a simple causal interpretation as synergy. Dose- and adherence-informed analyses will aid interpretation but cannot remove this structural difference. Formal interaction results will therefore remain exploratory and hypothesis-generating.

Strengths include implementation in real-world long-term care facilities, blinded outcome assessment, multiple active comparison arms, standardised fidelity monitoring, and clinically meaningful outcome domains. The six-month follow-up will provide information on maintenance of the primary outcomes and selected cognitive measures. The TIDieR-based supplementary description supports replication by specifying providers, materials, dose, progression, tailoring, and fidelity procedures.

The trial also has limitations. Participants and providers cannot be blinded, usual care may vary across facilities, and facility culture or staffing may influence adherence. The target sample is pragmatic and may be underpowered for small effects, many secondary outcomes, and formal exercise-by-cognitive-stimulation interactions. Attrition by the six-month follow-up may further reduce precision and introduce bias if loss to follow-up is differential; retention will therefore be reported by arm and addressed through mixed models and sensitivity analyses. Most secondary outcomes are assessed only at T0 and T1, so their long-term maintenance cannot be evaluated. No residents or public contributors were formally involved in designing the original protocol. The cognitive-stimulation component extrapolates evidence derived largely from people with dementia to residents without a major neurocognitive disorder, and its effectiveness in this population remains uncertain. Excluding residents unable to provide consent or follow instructions also limits generalisability to those with more severe cognitive impairment. Finally, the combined arm’s greater contact time precludes confirmatory mechanistic claims of synergy.

If the interventions demonstrate clinically meaningful benefits, the findings will inform scalable strategies to preserve mobility, cognition, and well-being in long-term care settings. Even modest effects may be relevant when programmes are safe, feasible, and integrated into routine care. Conversely, if single-component programmes provide benefits comparable to the combined programme, facilities with limited staffing may prioritise the component most aligned with local needs and resources. The trial will also provide useful information about recruitment, retention, intervention dose, adverse events, and outcome responsiveness for future confirmatory research and may help define realistic implementation pathways in long-term care.

## 4. Conclusions

This protocol presents a pragmatic, randomised trial whose primary objective is to estimate the effect of combined physical exercise plus cognitive stimulation versus usual care on executive function, functional mobility, and health-related quality of life at 12 weeks in older adults residing in long-term care. Secondary and interaction analyses will provide exploratory estimates of the isolated and combined components, while the six-month assessment will examine maintenance of prespecified outcomes. The findings may inform future confirmatory trials and feasible strategies for preserving mobility, cognition, and psychosocial well-being in long-term care.

## Figures and Tables

**Table 1:** Summary of intervention arms and comparator.

Arm	Core Content and Rationale	Dose and Delivery	Providers and Fidelity
Physical exercise	Multicomponent group programme targeting balance, strength, aerobic endurance, flexibility, and functional mobility; standardised session sequence with body weight, chairs, elastic bands, bottles, or ankle weights.	12 weeks; 3 sessions/week; approximately 60 min/session (50 min active plus transitions/rest); positive talk test; Borg CR10 initially 2–3 and progressing to 3–4; progression reviewed every 2 weeks.	Two physiotherapists trained in the protocol; attendance, safety events, and deviations recorded for each session.
Cognitive stimulation	Group activities targeting memory, attention, executive function, language, orientation, calculation, and visuospatial ability to promote cognitive engagement and psychosocial well-being.	12 weeks; 3 sessions/week; 60 min per session; progression from simpler to more complex tasks based on comprehension and performance.	Psychologist and sociocultural animator trained in the protocol; attendance and deviations recorded for each session.
Combined intervention	Sequential delivery of the physical exercise and cognitive stimulation programmes in the same visit to target complementary motor and cognitive domains.	12 weeks; 3 combined visits/week; each visit includes one exercise session immediately followed by one cognitive stimulation session.	Multidisciplinary team; component-specific attendance recorded separately for exercise and cognitive stimulation.
Usual care	Routine institutional activities without a standardised exercise or cognitive stimulation programme delivered by the study team.	Participants maintain routine activities during the 12-week study period; changes in usual care and co-interventions documented monthly.	Routine institutional staff; monthly monitoring logs used to document relevant co-interventions or activity changes.

**Table 2:** Outcomes and measurement instruments.

Domain	Outcome	Instrument/Measure	Metric	Time Points
Functional mobility	Primary	Timed Up and Go (TUG); dual-task TUG supportive	Time in seconds; lower is better	T0, T1, T2
Executive function	Primary	Torga version of the Stroop Colour-Word Test	Inhibitory-control index: correctly read words/s minus correctly named incongruent colours/s; lower is better	T0, T1, T2
Quality of life	Primary	EuroQol 5-Dimension 5-Level (EQ-5D-5L)	Index value primary; EQ visual analogue scale (EQ-VAS) supportive	T0, T1, T2
Balance	Secondary	Performance-Oriented Mobility Assessment (POMA/Tinetti)	0–28; higher is better	T0, T1
Strength and flexibility	Secondary	30-s Chair Stand; Arm Curl; Chair Sit-and-Reach; Back Scratch; dynamometry	Repetitions, cm, and kg as appropriate	T0, T1
Aerobic endurance	Secondary	2-Minute Step Test	Step count; higher is better	T0, T1
Executive function	Secondary	Trail Making Test (TMT); Digit Span	Time/standard scores as appropriate	T0, T1, T2
Global cognition	Secondary	Mini-Mental State Examination (MMSE)	Standard score	T0, T1
Sleep	Secondary	Pittsburgh Sleep Quality Index (PSQI)	0–21; lower is better	T0, T1
Psychological outcomes	Secondary	Geriatric Depression Scale (GDS); Satisfaction With Life Scale (SWLS); Psychological Well-Being Scales (PWBS)	Scale-specific	T0, T1
Function and activity	Secondary	Barthel Index; Global Physical Activity Questionnaire (GPAQ); pedometer	Index score, MET-min/week, steps/day	T0, T1

Abbreviations: EQ-5D-5L, EuroQol 5-Dimension 5-Level; EQ-VAS, EuroQol visual analogue scale; GDS, Geriatric Depression Scale; GPAQ, Global Physical Activity Questionnaire; MMSE, Mini-Mental State Examination; POMA, Performance-Oriented Mobility Assessment; PSQI, Pittsburgh Sleep Quality Index; PWBS, Psychological Well-Being Scales; SWLS, Satisfaction With Life Scale; TMT, Trail Making Test; TUG, Timed Up and Go.

**Table 3:** Schedule of enrolment, interventions, and assessments (SPIRIT).

Activity	Screening	Baseline (T0)	Post-Intervention (T1, 12 Weeks)	Follow-Up (T2, 6 Months)
Eligibility screening	X			
Informed consent		X		
Allocation		X		
Sociodemographic and clinical data		X		
Anthropometrics and vital signs		X	X	X
Functional mobility (TUG; dual-task TUG)		X	X	X
Executive function (Stroop; Trail Making Test; Digit Span)		X	X	X
Global cognition (MMSE)		X	X	
Balance (POMA/Tinetti)		X	X	
Strength and flexibility (Senior Fitness Test components; grip strength)		X	X	
Aerobic endurance (2-min step test)		X	X	
Quality of life (EQ-5D-5L index and EQ-VAS)		X	X	X
Sleep quality (PSQI)		X	X	
Psychological outcomes (GDS; SWLS; PWBS)		X	X	
Functional independence (Barthel Index)		X	X	
Physical activity (GPAQ; pedometer 8 days)		X	X	
Adverse events monitoring			X	X
Adherence/attendance logs			X	

**Table 4:** Summary of planned estimands and main analytical comparisons.

Comparison/ Estimand	Primary Endpoint	Planned Contrast	Analytical Approach	Remarks
Combined intervention vs. usual care	Stroop interference score at T1	Adjusted mean difference in change from baseline	Linear mixed-effects model	Principal efficacy comparison; Holm-adjusted primary family
Combined intervention vs. usual care	TUG time at T1	Adjusted mean difference in change from baseline	Linear mixed-effects model	Primary mobility domain; Holm-adjusted primary family
Combined intervention vs. usual care	EQ-5D-5L index at T1	Adjusted mean difference in change from baseline	Linear mixed-effects model	Primary quality-of-life domain; Holm-adjusted primary family
Exercise vs. usual care	Primary and secondary outcomes	Group-by-time contrasts	Mixed-effects models	Exploratory component-specific effects; FDR-controlled outcome families
Cognitive stimulation vs. usual care	Primary and secondary outcomes	Group-by-time contrasts	Mixed-effects models	Exploratory component-specific effects; FDR-controlled outcome families
Combined vs. exercise/combined vs. cognitive stimulation	Primary and secondary outcomes	Group-by-time contrasts	Mixed-effects models	Exploratory added-value comparisons; no confirmatory synergy claim
Exercise × cognitive stimulation interaction	Primary outcomes	Exercise-by-cognitive-stimulation-by-time interaction	Exploratory mixed-effects model	Hypothesis-generating; the trial is not powered for confirmatory interaction testing
Sensitivity analyses	Key outcomes	Per-protocol, adherence-informed, and missing-data analyses	Alternative model specifications/imputation	Supports robustness and interpretation

## Data Availability

No trial dataset is reported in this protocol manuscript because participant recruitment and data collection had not yet started at the time of submission. De-identified data generated during the trial may be made available from the corresponding author on reasonable request, subject to ethical, legal, and institutional constraints.

## Supplementary Materials

The following supporting information can be downloaded at: https://www.mdpi.com/article/10.3390/jcm15166327/s1, Supplementary Material S1: SPIRIT 2025 checklist; Supplementary Material S2: TIDieR intervention description; Supplementary Material S3: Portuguese participant information sheet and informed consent form with a full English translation.

## Abbreviations

ACSM, American College of Sports Medicine; AE, adverse event; BH, Benjamini–Hochberg; COM-B, capability, opportunity, motivation, and behaviour; EQ-5D-5L, EuroQol 5-Dimension 5-Level; EQ-VAS, EuroQol visual analogue scale; GDS, Geriatric Depression Scale; GPAQ, Global Physical Activity Questionnaire; ITT, intention-to-treat; MMSE, Mini-Mental State Examination; POMA, Performance-Oriented Mobility Assessment; PSQI, Pittsburgh Sleep Quality Index; PWBS, Psychological Well-Being Scales; RCT, randomised controlled trial; RPE, rating of perceived exertion; SPIRIT, Standard Protocol Items: Recommendations for Interventional Trials; SWLS, Satisfaction With Life Scale; TIDieR, Template for Intervention Description and Replication; TMT, Trail Making Test; TUG, Timed Up and Go.
