# Supplementary material for: Effects of Physical Exercise, Cognitive Stimulation, and Their Combination on Mobility, Cognitive Function, and Psychosocial Well-Being in Older Adults Residing in Long-Term Care: Protocol for a Randomised Controlled Trial
Source: J Clin Med. 2026 Aug 16;15(16):6327. doi: 10.3390/jcm15166327 (PMC13512956; doi:10.3390/jcm15166327)
Supplement: Supplementary file 1 [file jcm-15-06327-s001.zip › jcm-4456373-supplementary.pdf]

Supplementary Material S1

SPIRIT 2025 Checklist

Manuscript title: Effects of Physical Exercise, Cognitive Stimulation, and Their Combination on Mobility, Cognitive Function, and Psychosocial Well-Being in Older Adults Residing in Long-Term Care: Protocol for a Randomised Controlled Trial

Authors: Nuno Carvalho; Rocío Llamas-Ramos; Inés Llamas-Ramos

Corresponding author: Rocío Llamas-Ramos, rociollamas@usal.es

This checklist indicates where each applicable SPIRIT 2025 item is addressed in the manuscript. All references to trial status correspond to a protocol for which no resident had been approached, screened, consented, or enrolled before written institutional authorisation was completed for all four ERPI units.

Section/topic	Nº	SPIRIT 2025 item description	Where addressed in manuscript
**Administrative information - Title and structured summary**	1a	Title stating the trial design, population, and interventions, with identification as a protocol	Title page
**Administrative information - Title and structured summary**	1b	Structured summary of trial design and methods, including items from the WHO Trial Registration Data Set	Abstract; Sections 2.1 and 2.2
**Administrative information - Protocol version**	2	Version date and identifier	Title page (protocol version and date)
**Administrative information - Roles and responsibilities**	3a	Names, affiliations, and roles of protocol contributors	Title page; Author Contributions
**Administrative information - Roles and responsibilities**	3b	Name and contact information for the trial sponsor	Title page correspondence details; Funding
**Administrative information - Roles and responsibilities**	3c	Role of trial sponsor and funders in design, conduct, analysis, and reporting of trial	Funding; Author Contributions
**Administrative information - Roles and responsibilities**	3d	Composition, roles, and responsibilities of coordinating site, oversight groups, and trial teams, if applicable	Sections 2.1, 2.5, 2.6, and 2.11; Author Contributions; Funding
**Open science - Trial registration**	4	Name of trial registry, identifying number, URL, and date of registration	Section 2.2; ClinicalTrials.gov record (NCT07082504)
**Open science - Protocol and statistical analysis plan**	5	Where the trial protocol and statistical analysis plan can be accessed	Sections 2.9 and 2.10; Data Availability Statement; ClinicalTrials.gov record
**Open science - Data sharing**	6	Where and how de-identified participant data, statistical code, and materials will be accessible	Data Availability Statement
**Administrative information - Funding and conflicts of interest**	7a	Sources of funding and other support	Funding
**Administrative information - Funding and conflicts of interest**	7b	Financial and other conflicts of interest	Conflicts of Interest

Section/topic	Nº	SPIRIT 2025 item description	Where addressed in manuscript
**Administrative information - Dissemination policy**	8	Plans to communicate trial results to participants, professionals, the public, and relevant groups	Section 2.2; Supplementary Material 3
**Introduction - Background and rationale**	9a	Scientific background and rationale, including relevant studies examining benefits and harms for each intervention	Section 1; Section 2.11
**Introduction - Background and rationale**	9b	Explanation for choice of comparator	Section 1; Section 2.6.3
**Introduction - Objectives**	10	Specific objectives related to benefits and harms	Section 1 (final paragraph); Sections 2.7, 2.8, and 2.11
**Methods - Patient and public involvement**	11	Details of, or plans for, patient or public involvement in trial design, conduct, and reporting	Section 2.3
**Methods - Trial design**	12	Description of trial design including type of trial, allocation ratio, and framework	Section 2.1
**Methods - Trial setting**	13	Settings and locations where the trial will be conducted	Section 2.1
**Methods - Eligibility criteria**	14a	Eligibility criteria for participants	Section 2.4
**Methods - Eligibility criteria**	14b	Eligibility criteria for sites and for individuals who deliver the interventions, if applicable	Sections 2.4 and 2.6; Supplementary Material 2
**Methods - Intervention and comparator**	15a	Intervention and comparator details sufficient for replication, including how, when, and by whom they will be administered	Section 2.6; Table 1; Supplementary Material 2
**Methods - Intervention and comparator**	15b	Criteria for discontinuing or modifying allocated intervention/comparator for a participant	Sections 2.4, 2.6.1, and 2.11
**Methods - Intervention and comparator**	15c	Strategies to improve and monitor adherence to intervention/comparator protocols	Sections 2.6 and 2.9; Supplementary Material 2
**Methods - Intervention and comparator**	15d	Concomitant care permitted or prohibited during the trial	Section 2.6.3
**Methods - Outcomes**	16	Primary and secondary outcomes, including measurement variable, analysis metric, aggregation method, and time point	Sections 2.7, 2.8, and 2.10; Tables 2-4
**Methods - Harms**	17	How harms are defined and assessed	Section 2.11
**Methods - Participant timeline**	18	Schedule of enrolment, interventions, assessments, and visits; schematic diagram recommended	Section 2.1; Table 3; Figure 1
**Methods - Sample size**	19	How sample size was determined, including assumptions	Section 2.8
**Methods - Recruitment**	20	Strategies for achieving adequate participant enrolment to reach target sample size	Section 2.4
**Methods - Assignment of interventions: sequence generation**	21a	Who will generate the random allocation sequence and method used	Section 2.5
**Methods - Assignment of interventions: sequence generation**	21b	Type of randomisation and details of stratification or planned restrictions, if applicable	Section 2.5
**Methods - Allocation concealment mechanism**	22	Mechanism to implement the random allocation sequence and conceal it until assignment	Section 2.5

Section/topic	Nº	SPIRIT 2025 item description	Where addressed in manuscript
**Methods - Implementation**	23	Whether personnel who enrol and assign participants will have access to the allocation sequence	Section 2.5
**Methods - Blinding**	24a	Who will be blinded after assignment to interventions	Section 2.5
**Methods - Blinding**	24b	How blinding will be achieved, if blinded, including similarity of interventions	Section 2.5
**Methods - Blinding**	24c	Circumstances and procedures for unblinding, if applicable	Section 2.5 (no planned assessor unblinding)
**Methods - Data collection**	25a	Plans for assessment and collection of trial data, data quality processes, and description of instruments with reliability/validity if known	Section 2.7; Tables 2 and 3; Section 2.9
**Methods - Data collection**	25b	Plans to promote retention and complete follow-up, including outcome data for participants who discontinue or deviate from protocols	Sections 2.4, 2.9, and 2.12
**Methods - Data management**	26	Plans for data entry, coding, security, storage, and quality processes	Section 2.9
**Methods - Statistical methods**	27a	Statistical methods for comparing groups for primary and secondary outcomes, including harms	Section 2.10; Table 4; Section 2.11
**Methods - Statistical methods**	27b	Definition of who will be included in each analysis and in which group	Section 2.10
**Methods - Statistical methods**	27c	How missing data will be handled in the analysis	Section 2.10
**Methods - Statistical methods**	27d	Methods for additional analyses, such as subgroup and sensitivity analyses	Section 2.10; Table 4
**Methods - Data monitoring committee**	28a	Data monitoring committee composition, role, independence, conflicts, and charter; or explanation why a DMC is not needed	Section 2.11
**Methods - Data monitoring committee**	28b	Interim analyses and stopping guidelines, including access to interim results and decision authority	Section 2.11
**Methods - Trial monitoring**	29	Frequency and procedures for monitoring trial conduct, or explanation if no monitoring	Sections 2.9 and 2.11
**Ethics - Research ethics approval**	30	Plans for seeking research ethics committee/institutional review board approval	Section 2.2; Institutional Review Board Statement
**Ethics - Protocol amendments**	31	Plans for communicating important protocol modifications to relevant parties	Section 2.9
**Ethics - Consent or assent**	32a	Who will obtain informed consent or assent and how	Section 2.2; Supplementary Material 3
**Ethics - Consent or assent**	32b	Additional consent provisions for collection and use of participant data and biological specimens in ancillary studies, if applicable	Section 2.2 (no biological specimens); Supplementary Material 3
**Ethics - Confidentiality**	33	How personal information will be collected, shared, and maintained to protect confidentiality	Sections 2.2 and 2.9; Data Availability Statement; Supplementary Material 3
**Ethics - Ancillary and post-trial care**	34	Ancillary and post-trial care and compensation for those who suffer harm from trial participation, if applicable	Section 2.11; Supplementary Material 3

Supplementary Material S2

TIDieR Intervention Description

Manuscript title: Effects of Physical Exercise, Cognitive Stimulation, and Their Combination on Mobility, Cognitive Function, and Psychosocial Well-Being in Older Adults Residing in Long-Term Care: Protocol for a Randomised Controlled Trial

Authors: Nuno Carvalho; Rocío Llamas-Ramos; Inés Llamas-Ramos

Corresponding author: Rocío Llamas-Ramos, rociollamas@usal.es

TIDieR items are provided for each planned trial arm to support intervention replicability. Because no resident had been approached, screened, consented, or enrolled before institutional authorisation, all delivery statements are expressed prospectively.

	Description	Arm A: Exercise	Arm B: Cognitive stimulation	Arm C: Combined	Control: Usual care
**1**	Provide the name or phrase that describes the intervention	Moderate-intensity multicomponent physical exercise programme	Group cognitive stimulation programme	Combined physical exercise plus cognitive stimulation	Usual care (control)
**2**	Rationale, theory, or goal of the elements essential to the intervention	Improve mobility-related function, strength, balance, and flexibility; reduce fall risk through progressive balance and functional training.	Enhance/maintain cognitive performance, with emphasis on attention and executive functions, and support mental well-being.	Potentially improve motor and cognitive outcomes by addressing both domains within the same programme.	Provides comparator reflecting routine activities without a standardised exercise/cognitive programme.
**3**	Physical or informational materials used	Standardised chairs (~46 cm), timers/stopwatches, elastic bands, body weight, water bottles/hand weights, ankle weights, cones/markers, and step platforms as available; Borg CR10 and session control sheets.	Cognitive task materials (paper/pencils, cards, printed worksheets, puzzles/riddles/brain teasers), orientation materials; computer materials if available.	Materials used in both the exercise and cognitive stimulation programmes, including Borg CR10 and session control sheets.	Not applicable (routine institutional resources).

	Description	Arm A: Exercise	Arm B: Cognitive stimulation	Arm C: Combined	Control: Usual care
**4**	Procedures, activities, and/or processes used	Each session comprises 5 min warm-up, 10 min balance, 20 min resistance/functional exercise, 10 min aerobic/mobility work, and 5 min cool-down/flexibility, with approximately 10 min available for transitions and rest. Core exercises include sit-to-stand, knee extension, plantar flexion, hip abduction, upper-limb pull/elbow flexion, and step practice.	Structured group activities targeting memory, attention, executive function, language, temporal-spatial orientation, calculation, and visuospatial skills (games, riddles, puzzles, reasoning/problem-solving exercises).	Both programmes are delivered sequentially during the same visit: the specified physical exercise session followed immediately by cognitive stimulation.	Routine non-standardised activities such as handicrafts/manual activities, games, and short indoor walks; no structured, standardised programme.
**5**	Expertise, background, and training of intervention providers	Will be delivered by two physiotherapists trained in the study protocol and safety procedures.	Will be delivered by a psychologist and a sociocultural animator trained in the study protocol.	Will be delivered by a multidisciplinary team trained in the study protocol and safety procedures.	Usual institutional staff.
**6**	Modes of delivery (e.g., face-to-face, group)	Face-to-face, small-group supervised sessions.	Face-to-face, small-group sessions.	Face-to-face, small-group sessions.	As routinely provided within each facility.
**7**	Type(s) of location(s) where intervention will occur	Four ERPI units operated by three non-profit private social solidarity providers in the district of Viseu, Portugal; written institutional authorisation has been obtained from all four units, and provider identities are withheld from the public article at their request.	Four ERPI units operated by three non-profit private social solidarity providers in the district of Viseu, Portugal; written institutional authorisation has been obtained from all four units, and provider identities are withheld from the public article at their request.	Four ERPI units operated by three non-profit private social solidarity providers in the district of Viseu, Portugal; written institutional authorisation has been obtained from all four units, and provider identities are withheld from the public article at their request.	Four ERPI units operated by three non-profit private social solidarity providers in the district of Viseu, Portugal; written institutional authorisation has been obtained from all four units, and provider identities are withheld from the public article at their request.
**8**	Number of sessions, schedule, duration, intensity/dose	12 weeks; 3 sessions/week; ~60 min scheduled (~50 min active exercise plus ~10 min transitions/rest). Weeks 1–2: 1 set of 8–10 repetitions; weeks 3–6: 2 sets of 8–12; weeks 7–12: 2–3 sets of 8–12. A positive talk test is maintained; Borg CR10 target is 2–3 initially and 3–4 as tolerated.	12 weeks; 3 sessions/week; 60 min per session. Progression from simpler tasks (to establish understanding and engagement) to more complex tasks over time.	12 weeks; 3 combined visits/week. Each visit includes the exercise dose specified for Arm A, followed by one 60-min cognitive stimulation session.	During the 12-week study period, participants will maintain routine activities.

	Description	Arm A: Exercise	Arm B: Cognitive stimulation	Arm C: Combined	Control: Usual care
**9**	Planned personalisation or adaptation	Exercises are adapted to individual functional capacity. Every 2 weeks, after two consecutive safely completed sessions, one variable at a time may be progressed in the following sequence: repetitions up to 12, then the smallest available resistance increment, task complexity, or aerobic duration, while maintaining the target talk test and Borg CR10 response.	Task difficulty will be adapted to participant comprehension and performance while maintaining standardised domains and session goals.	Adaptations follow the criteria for each component. Exercise progression follows the same sequence used for Arm A: repetitions up to 12, then the smallest available resistance increment, task complexity, or aerobic duration. Cognitive-task difficulty is adapted to comprehension and performance.	Not applicable.
**10**	Changes made during the course of the study	No planned modifications; any deviations will be recorded in session logs.	No planned modifications; any deviations will be recorded in session logs.	No planned modifications; any deviations will be recorded in session logs.	Not applicable.
**11**	Planned measures to assess and maintain fidelity	Session control sheet documenting attendance, exercises, sets, repetitions, resistance, task complexity, aerobic duration, talk-test response, Borg CR10, symptoms, adverse events, and progression decisions; standardised structure and provider training.	Session control sheet including attendance and occurrence log; standardised facilitation approach; provider training.	Control sheets for both components, including exercise dose (sets, repetitions, resistance, task complexity, and aerobic duration), talk-test response, Borg CR10, attendance, occurrences, deviations, and progression decisions; standardised procedures and provider training.	Facilities will be asked to notify the research team if participants initiate physiotherapy, structured sports, or similar activities during the study; deviations will be recorded.
**12**	Extent to which intervention is delivered as planned	To be reported in the results manuscript; adherence will be summarised from attendance records and session logs.	To be reported in the results manuscript; adherence will be summarised from attendance records and session logs.	To be reported in the results manuscript; adherence will be summarised from attendance and logs.	Not applicable (no active intervention); any co-interventions will be documented.

Supplementary Material S3

Participant Information Sheet and Informed Consent Form (Portuguese Version)

Manuscript title: Effects of Physical Exercise, Cognitive Stimulation, and Their Combination on Mobility, Cognitive Function, and Psychosocial Well-Being in Older Adults Residing in Long-Term Care: Protocol for a Randomised Controlled Trial

Authors: Nuno Carvalho; Rocío Llamas-Ramos; Inés Llamas-Ramos

Corresponding author: Rocío Llamas-Ramos, rociollamas@usal.es

The following material presents the Portuguese participant information sheet, informed consent form, withdrawal form, and privacy policy planned for use in the trial, followed by a complete English translation supplied for accessibility to an international readership. No resident was approached, screened, consented, or enrolled before institutional authorisation was completed.

FICHA DE INFORMAÇÃO AOS PARTICIPANTES E DE CONSENTIMENTO INFORMADO PARA PROJETOS DE INVESTIGAÇÃO COM PESSOAS COM PLENA CAPACIDADE

Título: Impacto de Programas de Exercício Físico, Estimulação Cognitiva e da sua Combinação na Mobilidade, Função Cognitiva e Bem-estar Psicossocial em Idosos Institucionalizados.

I.P.: Inés Llamas Ramos

Investigador doutorando / coordenação operacional: Nuno Carvalho

Introdução

Foi convidado(a) a participar num estudo de investigação. Leia atentamente as informações que se seguem para esclarecer quaisquer dúvidas. Coloque quaisquer questões ao(à) investigador(a) deste estudo caso algo não lhe esteja claro ou se pretender obter mais informações.

Este projeto foi aprovado favoravelmente pelo Comité de Ética da Universidade de Salamanca e cumpre as recomendações éticas da Declaração de Helsínquia.

Este estudo está relacionado com o envelhecimento da população, um desafio crescente, sobretudo no caso de pessoas idosas que residem em lares ou instituições, e não nas suas casas. Esta condição está associada a maior perda de autonomia e a um declínio mais rápido das capacidades físicas e cognitivas. A permanência numa instituição pode reduzir a estimulação física e mental, afetando negativamente a qualidade de vida e promovendo um aumento da dependência. Para atenuar estes efeitos, é essencial adotar estratégias eficazes. O exercício físico pode melhorar a força, o equilíbrio e a mobilidade, reduzindo o risco de quedas e promovendo a funcionalidade. Por outro lado, a estimulação cognitiva pode ajudar a preservar capacidades mentais e a retardar o declínio associado ao envelhecimento. A combinação de ambas as estratégias tem demonstrado potencial para produzir benefícios mais amplos do que intervenções isoladas.

No entanto, embora os resultados de estudos anteriores sugiram benefícios consideráveis, continua a existir a necessidade de novas investigações que aprofundem os mecanismos subjacentes e as melhores formas de implementar estas estratégias em pessoas que não vivem no seu domicílio. Estudos adicionais poderão contribuir para o desenvolvimento de programas mais eficazes e personalizados, com o objetivo de melhorar a qualidade de vida e reduzir a dependência dos idosos institucionalizados.

Objetivo do estudo

O objetivo do estudo é analisar a eficácia de três tipos de intervenções em idosos institucionalizados: um programa de exercício físico, um programa de estimulação cognitiva e um programa que combina ambas

as estratégias. A investigação focar-se-á na avaliação dos efeitos destas intervenções na força, flexibilidade, equilíbrio e mobilidade funcional, bem como em variáveis cognitivas e emocionais, incluindo a função executiva (planeamento, tomada de decisões, resolução de problemas...), o estado mental, a qualidade do sono e a qualidade de vida.

Procedimentos

O projeto será desenvolvido em três fases, com uma intervenção de 12 semanas, seguida de um acompanhamento de 6 meses. As avaliações serão efetuadas em três momentos: antes da intervenção (pré-intervenção), no final da intervenção (12 semanas após o início) e 6 meses depois da conclusão do programa.

As avaliações baseiam-se em instrumentos previamente validados. Estas avaliações incluem questionários, assim como testes físicos e de mobilidade que avaliam aspetos essenciais da saúde dos participantes. Será avaliada a mobilidade funcional, o equilíbrio, o medo de cair, a força, a flexibilidade e a resistência aeróbica através de vários testes físicos. Além disso, será avaliado o estado mental, bem como diferentes tarefas, nomeadamente o planeamento, a tomada de decisões e a resolução de problemas através de questionários específicos. Serão também avaliados a qualidade de vida, o sono e a atividade física, utilizando instrumentos concebidos para captar estes aspetos. Por último, serão recolhidos dados sobre a funcionalidade, a dor e as caraterísticas sociodemográficas e clínicas dos participantes.

Os participantes no estudo, com 65 anos de idade ou mais e a residir num lar de idosos há mais de três meses, serão atribuídos aleatoriamente a um dos quatro grupos: um grupo realizará exercício físico, outro participará em estimulação cognitiva, um terceiro combinará ambas as abordagens, enquanto o quarto grupo servirá como grupo de controlo, mantendo os cuidados e atividades habituais.

A intervenção terá uma duração de 12 semanas, com três sessões semanais. O grupo de exercício físico participará em sessões de aproximadamente 60 minutos, incluindo aquecimento, fase principal e retorno à calma, com intensidade moderada, conforme as recomendações da Organização Mundial de Saúde (OMS) e do American College of Sports Medicine (ACSM). As atividades incluirão exercícios destinados a melhorar o equilíbrio, a força muscular e a mobilidade, sob a orientação de fisioterapeutas treinados no protocolo.

O grupo de estimulação cognitiva realizará atividades focadas na melhoria da memória, atenção, funções executivas e outras capacidades cognitivas, incluindo jogos, enigmas, exercícios de raciocínio e tarefas de resolução de problemas, sob supervisão de um psicólogo e de uma animadora sociocultural treinados no protocolo.

Por último, o grupo combinado realizará ambos os programas na mesma visita, com a sessão de exercício físico seguida de estimulação cognitiva.

Princípio da não maleficência: Riscos e obstáculos

A participação neste estudo envolve riscos mínimos. Poderá ocorrer cansaço, desconforto muscular ligeiro, tontura transitória, receio de queda ou desconforto associado à realização de tarefas físicas ou cognitivas. Todas as sessões serão supervisionadas por profissionais treinados e serão interrompidas sempre que surjam sintomas ou preocupações de segurança. Se necessário, o participante será encaminhado para os cuidados institucionais ou médicos adequados.

Princípio da autonomia e benefícios da participação: participação e desistência voluntárias

O participante é livre de decidir se pretende ou não participar neste estudo, sendo a sua participação inteiramente voluntária. Se decidir participar, mantém o direito de se retirar a qualquer momento, sem necessidade de justificar a decisão e sem qualquer penalização ou consequências negativas para si. A sua

decisão de se retirar não o afetará de forma alguma. Se decidir participar, deverá empenhar-se em cumprir, da melhor forma possível, as indicações fornecidas pela equipa de investigação.

Direito à informação

Embora possa não beneficiar diretamente da sua participação neste estudo, estará a contribuir para o desenvolvimento do conhecimento científico em áreas da geriatria e gerontologia, bem como da fisioterapia e do exercício.

[ ] Sim, gostaria de receber os resultados gerais da investigação.

[ ] Não pretendo receber informações.

A informação será enviada num envelope fechado para a instituição (lar de idosos).

Medidas de confidencialidade e segurança

Todas as informações recolhidas durante este estudo serão tratadas de forma estritamente confidencial, em conformidade com a política de privacidade (ver anexo).

Será implementado um sistema de codificação/pseudonimização dos dados para reduzir o risco de identificação dos participantes. Em circunstância alguma os consentimentos assinados que identificam os participantes serão reunidos com os questionários ou demais informações utilizadas na análise. Na utilização dos resultados do estudo para fins de ensino, investigação e/ou publicação, será sempre respeitada a proteção dos dados pessoais, de modo a garantir que os participantes não sejam identificados ou identificáveis.

Caso os resultados do estudo sejam publicados em revistas científicas, em nenhuma circunstância serão divulgados os dados pessoais dos participantes nesta investigação.

É importante que não divulgue as caraterísticas dos procedimentos nem os objetivos deste estudo até que toda a investigação esteja concluída.

Dados de contacto da equipa de investigação:

Nome: Nuno Carvalho

Telefone: +351 932364371

Nome: Inés Llamas Ramos

Telefone: +34 663005052

CONSENTIMENTO INFORMADO PARA PESSOAS COM PLENA CAPACIDADE

Título: Impacto de Programas de Exercício Físico, Estimulação Cognitiva e da sua Combinação na Mobilidade, Função Cognitiva e Bem-estar Psicossocial em Idosos Institucionalizados.

Eu (Nome, Apelido e Nº documento de identificação) ________________________________

Pude fazer perguntas sobre o estudo.

Falei com o(a) Investigador(a) ______________________

Recebi informações suficientes sobre o estudo.

Li a ficha de informação que me foi fornecida.

Fui informado sobre a forma como os meus dados serão tratados.

Compreendo que a minha participação é voluntária.

Compreendo que posso retirar-me do estudo:

1º Sempre que quiser.

2º Sem ter de dar explicações.

3º Sem quaisquer repercussões negativas.

Aceito, de forma voluntária, participar no projeto e autorizo a utilização de todas as informações obtidas. Tomei conhecimento de que receberei uma cópia assinada do presente consentimento informado.

Assinatura do participante Data

Nome e assinatura do investigador Data

REVOGAÇÃO DO CONSENTIMENTO

Revogo o consentimento dado no dia ____________ para participar no projeto intitulado "Impacto de Programas de Exercício Físico, Estimulação Cognitiva e da sua Combinação na Mobilidade, Função Cognitiva e Bem-estar Psicossocial em Idosos Institucionalizados." e, para que fique registado, assino a presente revogação.

Em ____________________ , no dia ________ de ________________________ de 20___.

Assinatura do participante Data

Nome e assinatura do investigador Data

POLÍTICA DE PRIVACIDADE

Quem processa os seus dados?

A pessoa responsável pelo tratamento dos seus dados é:

Universidade de Salamanca

C.I.F. Q3718001E

C/ Patio de las Escuelas Menores, nº 1

C.P. 37008, Salamanca

Como pode contactar o nosso responsável pela proteção de dados?

O responsável pela proteção de dados é a pessoa encarregada de supervisionar o cumprimento das regras de proteção de dados e por prestar apoio sempre que necessário. Se tiver alguma dúvida ou questão sobre a forma como tratamos os seus dados, pode contactar o responsável pela proteção de dados: dpd@usal.es

Com que finalidade tratamos os seus dados? Por que motivo e com que fundamento legal tratamos os seus dados?

Os seus dados serão tratados com a finalidade de gerir a sua participação no projeto de investigação. Os seus dados serão tratados em conformidade com:

O seu consentimento (artigo 6.º n.º 1, alínea a) do RGPD), para participar no projeto, bem como a eventual publicação dos resultados, nomeadamente no que se refere a referências biográficas cuja divulgação possa ser necessária.

Cumprimento de uma missão de interesse público ou no exercício de autoridade pública conferida ao responsável pelo tratamento de dados (artigo 6.º, n.º 1, alínea e) do RGPD)

Com quem partilhamos os seus dados?

- Os dados só serão comunicados, sem necessidade de consentimento, a pedido das autoridades competentes.

Nesses casos, antes de disponibilizar os dados a terceiros, a Universidade assegura que essas autoridades solicitem e acedam aos dados em conformidade com a lei.

Durante quanto tempo conservaremos os dados?

▪ Os dados serão utilizados ao longo de toda a investigação até à eventual elaboração de um relatório ou à publicação dos resultados da investigação.

▪ As informações serão mantidas devidamente bloqueadas durante os períodos adicionais necessários para a prescrição de eventuais responsabilidades legais.

▪ As informações de valor histórico serão conservadas indefinidamente, mediante aprovação prévia da Comissão de Expurgo, de acordo com o disposto na Lei 16/1985, de 25 de junho, do Património Histórico Espanhol, e na legislação específica aplicável.

Como protegemos as informações?

Enquanto administração pública, aplicamos as medidas técnicas e organizacionais estabelecidas pelo Regime de Segurança Nacional. Estas incluem uma série de recomendações destinadas a garantir a segurança dos sistemas de informação, prevenindo o roubo, alteração ou acessos não autorizados aos dados.

Em caso de subcontratação de serviços, exigiremos e asseguraremos que o responsável pelo tratamento aplique medidas equivalentes às previstas no Regime de Segurança Nacional.

Quais são os direitos dos participantes?

A fim de manter sempre o controlo sobre os seus dados, tem o direito de aceder às suas informações pessoais, bem como o direito de solicitar a retificação desses dados incorretos ou, se for caso disso, de solicitar a sua eliminação ou cancelamento. Em determinadas circunstâncias, e por motivos relacionados com a sua situação particular, pode opor-se ao tratamento dos seus dados. Do mesmo modo, pode exercer o seu direito de limitar o tratamento das suas informações pessoais, solicitando a sua conservação, bem como a portabilidade das mesmas.

O exercício dos direitos é pessoal, pelo que é necessário identificá-lo de forma inequívoca. Pode exercer os seus direitos de duas formas:

▪ Enviando um e-mail para o endereço indicado.

Para o fazer, utilize este contacto: dpd@usal.es. Apenas serão atendidos os pedidos efetuados a partir de contas de e-mail fornecidas pela Universidade de Salamanca ou que constem nas nossas bases de dados, após identificação do respetivo titular.

▪ Através da entrega de um requerimento no nosso Registo ou por correio postal dirigido a:

Secretaría General

Universidad de Salamanca.

C.I.F. Q3718001E

C/ Patio de las Escuelas Menores, nº 1

C.P. 37008, Salamanca

Deve apresentar a seguinte documentação comprovativa:

▪ Comprovação da identidade do interessado mediante apresentação de um documento válido, como o documento de identificação ou passaporte.

▪ Nome e apelidos do interessado ou, quando aplicável, da pessoa que a representa, bem como o documento que comprove essa representação.

▪ O pedido apresentado.

▪ Morada para efeitos de notificação, data e assinatura do requerente.

▪ Documentos comprovativos do pedido que está a fazer, quando aplicável.

▪ Em caso de retificação ou eliminação, indicação dos dados a retificar ou a eliminar, bem como o motivo que a justifica.

Quem garante os direitos dos participantes? A quem se pode queixar?

Caso pretenda apresentar uma queixa ou obter mais informações sobre a regulamentação do tratamento de dados pessoais em Espanha, a autoridade competente é a Agencia Española de Protección de Datos (Agência Espanhola de Proteção de Dados).

This English version is supplied for accessibility to an international readership. Participants will receive the Portuguese-language documents reproduced above.

PARTICIPANT INFORMATION SHEET AND INFORMED CONSENT FORM FOR RESEARCH PROJECTS INVOLVING ADULTS WITH FULL DECISION-MAKING CAPACITY

Title: Effects of Physical Exercise Programmes, Cognitive Stimulation, and Their Combination on Mobility, Cognitive Function, and Psychosocial Well-Being in Older Adults Residing in Long-Term Care.

Principal Investigator: Inés Llamas Ramos

Doctoral researcher / operational coordinator: Nuno Carvalho

Introduction

You have been invited to take part in a research study. Please read the following information carefully to clarify any questions you may have. Ask the study researcher any questions if anything is unclear or if you would like further information.

This project received a favourable opinion from the Research Ethics Committee of the University of Salamanca and complies with the ethical recommendations of the Declaration of Helsinki.

This study concerns population ageing, a growing challenge, particularly for older people who live in care homes or institutions rather than in their own homes. This situation is associated with greater loss of autonomy and a faster decline in physical and cognitive abilities. Living in an institution may reduce physical and mental stimulation, negatively affecting quality of life and increasing dependence. Effective strategies are therefore essential to mitigate these effects. Physical exercise can improve strength, balance, and mobility, reduce the risk of falls, and promote functional ability. Cognitive stimulation may help preserve mental abilities and delay age-related decline. Combining both strategies has shown potential to produce broader benefits than either intervention alone.

However, although previous studies suggest considerable benefits, further research is needed to clarify the underlying mechanisms and the best ways to implement these strategies in people who do not live in their own homes. Additional studies may contribute to developing more effective and personalised programmes aimed at improving quality of life and reducing dependence among older people residing in long-term care.

Study objective

The aim of this study is to analyse the effectiveness of three types of intervention in older people residing in long-term care: a physical exercise programme, a cognitive stimulation programme, and a programme combining both strategies. The study will assess the effects of these interventions on strength, flexibility, balance, and functional mobility, as well as on cognitive and emotional variables, including executive function (planning, decision-making, problem-solving, etc.), mental status, sleep quality, and quality of life.

Procedures

The project will be conducted in three phases, with a 12-week intervention followed by a 6-month follow-up. Assessments will take place at three time points: before the intervention (pre-intervention), at the end of the intervention (12 weeks after it begins), and 6 months after completion of the programme.

The assessments use previously validated instruments. They include questionnaires and physical and mobility tests that evaluate key aspects of participants' health. Functional mobility, balance, fear of falling, strength, flexibility, and aerobic endurance will be assessed using several physical tests. Mental status and tasks involving planning, decision-making, and problem-solving will also be evaluated using

specific questionnaires. Quality of life, sleep, and physical activity will be assessed with instruments designed to capture these dimensions. Finally, information on functional ability, pain, and participants' sociodemographic and clinical characteristics will be collected.

Study participants, aged 65 years or older and living in a care home for more than three months, will be randomly allocated to one of four groups: one group will undertake physical exercise, another will participate in cognitive stimulation, a third will combine both approaches, and the fourth will serve as a control group and continue usual care and activities.

The intervention will last 12 weeks, with three sessions per week. The physical exercise group will take part in sessions lasting approximately 60 minutes, including a warm-up, a main phase, and a cool-down, at moderate intensity in accordance with recommendations from the World Health Organization (WHO) and the American College of Sports Medicine (ACSM). Activities will include exercises designed to improve balance, muscle strength, and mobility, under the guidance of physiotherapists trained in the protocol.

The cognitive stimulation group will undertake activities focused on improving memory, attention, executive functions, and other cognitive abilities, including games, riddles, reasoning exercises, and problem-solving tasks, supervised by a psychologist and a sociocultural activity facilitator trained in the protocol.

Finally, the combined group will undertake both programmes during the same visit, with the physical exercise session followed by cognitive stimulation.

Principle of non-maleficence: risks and possible difficulties

Participation in this study involves minimal risks. Participants may experience tiredness, mild muscle discomfort, transient dizziness, fear of falling, or discomfort associated with physical or cognitive tasks. All sessions will be supervised by trained professionals and will be stopped whenever symptoms or safety concerns arise. If necessary, the participant will be referred to appropriate institutional or medical care.

Principle of autonomy and benefits of participation: voluntary participation and withdrawal

You are free to decide whether or not to take part in this study, and participation is entirely voluntary. If you decide to participate, you retain the right to withdraw at any time without having to give a reason and without any penalty or negative consequences. Your decision to withdraw will not affect you in any way. If you decide to take part, you should make every reasonable effort to follow the instructions provided by the research team.

Right to information

Although you may not benefit directly from taking part in this study, you will contribute to the development of scientific knowledge in geriatrics and gerontology, as well as in physiotherapy and exercise.

☐ Yes, I would like to receive the general research results.

☐ No, I do not wish to receive information.

The information will be sent in a sealed envelope to the institution (care home).

Confidentiality and security measures

All information collected during this study will be treated as strictly confidential, in accordance with the privacy policy (see annex).

A data-coding/pseudonymisation system will be implemented to reduce the risk of identifying participants. Signed consent forms that identify participants will never be stored together with questionnaires or other information used in the analysis. When study findings are used for teaching, research, and/or publication, personal data will always be protected so that participants cannot be identified or rendered identifiable.

If the study results are published in scientific journals, participants' personal data will not be disclosed under any circumstances.

It is important that you do not disclose the characteristics of the procedures or the objectives of this study until the entire investigation has been completed.

Research team contact details

Name: Nuno Carvalho

Telephone: +351 932364371

Name: Inés Llamas Ramos

Telephone: +34 663005052

INFORMED CONSENT FOR ADULTS WITH FULL DECISION-MAKING CAPACITY

Title: Effects of Physical Exercise Programmes, Cognitive Stimulation, and Their Combination on Mobility, Cognitive Function, and Psychosocial Well-Being in Older Adults Residing in Long-Term Care.

I (full name and identification document number) ________________________________

I have had the opportunity to ask questions about the study.

I spoke with the researcher ________________________________

I received sufficient information about the study.

I read the information sheet provided to me.

I was informed about how my data will be processed.

I understand that my participation is voluntary.

I understand that I may withdraw from the study:

1. At any time.
2. Without having to give an explanation.
3. Without any negative consequences.

I voluntarily agree to take part in the project and authorise the use of all information obtained. I understand that I will receive a signed copy of this informed consent form.

Participant's signature Date

Researcher's name and signature Date

WITHDRAWAL OF CONSENT

I withdraw the consent given on _________________ to participate in the project entitled "Effects of Physical Exercise Programmes, Cognitive Stimulation, and Their Combination on Mobility, Cognitive Function, and Psychosocial Well-Being in Older Adults Residing in Long-Term Care" and sign this withdrawal form so that my decision is formally recorded.

At ____________________, on the _____ day of _______________________, 20___.

Participant's signature Date

Researcher's name and signature Date

PRIVACY POLICY

Who processes your data?

The data controller responsible for processing your data is:

University of Salamanca

Tax identification number (C.I.F.): Q3718001E

C/ Patio de las Escuelas Menores, no. 1

Postcode 37008, Salamanca

How can you contact our Data Protection Officer?

The Data Protection Officer is responsible for overseeing compliance with data-protection rules and for providing assistance whenever necessary. If you have any doubts or questions about how we process your data, you may contact the Data Protection Officer at dpd@usal.es.

For what purpose do we process your data, and on what legal basis?

Your data will be processed to manage your participation in the research project. Your data will be processed on the following bases:

- Your consent (Article 6(1)(a) of the GDPR) to participate in the project and for the possible publication of the results, particularly with regard to biographical references whose disclosure may be necessary.
- Performance of a task carried out in the public interest or in the exercise of official authority vested in the data controller (Article 6(1)(e) of the GDPR).

With whom do we share your data?

- The data will be disclosed without requiring consent only when requested by competent authorities.

In such cases, before making data available to third parties, the University will ensure that those authorities request and access the data in accordance with the law.

How long will we retain the data?

- The data will be used throughout the research until a report is prepared or the research findings are published, as applicable.
- The information will be kept duly restricted for any additional periods required until potential legal liabilities become time-barred.
- Information of historical value will be retained indefinitely, subject to prior approval by the Records Appraisal and Disposal Committee, in accordance with Spanish Law 16/1985 of 25 June on Spanish Historical Heritage and the applicable specific legislation.

How do we protect the information?

As a public administration, we apply the technical and organisational measures established by the Spanish National Security Framework. These include recommendations designed to ensure the security of information systems and prevent theft, alteration, or unauthorised access to data.

If services are outsourced, we will require and ensure that the processor applies measures equivalent to those provided for in the Spanish National Security Framework.

What are participants' rights?

To retain control over your data at all times, you have the right to access your personal information and to request correction of inaccurate data or, where appropriate, its erasure or deletion. In certain circumstances and for reasons related to your particular situation, you may object to the processing of your data. You may also exercise your right to restrict the processing of your personal information, request that it be retained, and exercise your right to data portability.

These rights must be exercised personally, so you must be identified unequivocally. You may exercise your rights in either of the following ways:

- By sending an email to the address indicated below.

For this purpose, use dpd@usal.es. Only requests sent from email accounts provided by the University of Salamanca or recorded in our databases will be processed, after the account holder has been identified.

- By submitting a request through our Registry or by post to:

General Secretariat

University of Salamanca

Tax identification number (C.I.F.): Q3718001E

C/ Patio de las Escuelas Menores, no. 1

Postcode 37008, Salamanca

You must provide the following supporting documentation:

- Proof of the data subject's identity by presenting a valid document, such as an identity card or passport.
- The data subject's full name or, where applicable, the full name of the representative, together with a document evidencing that representation.
- The request being submitted.
- Address for notification purposes, date, and the applicant's signature.
- Documents supporting the request, where applicable.
- For correction or erasure, identification of the data to be corrected or erased and the reason supporting the request.

Who safeguards participants' rights, and to whom can you complain?

If you wish to lodge a complaint or obtain further information about the rules governing personal-data processing in Spain, the competent authority is the Agencia Española de Protección de Datos (Spanish Data Protection Agency).
